# *Pseudomonas aeruginosa* keratitis: passive immunotherapy with antibodies raised against divalent flagellin

**DOI:** 10.22038/ijbms.2018.31499.7643

**Published:** 2019-01

**Authors:** Pariya Mahin Samadi, Parmida Gerami, Ali Elmi, Korosh Khanaki, Sobhan Faezi

**Affiliations:** 1 Department of Microbiology, Pharmaceutical Sciences Branch, Islamic Azad University, Tehran, Iran; 2 Department of Microbiology, Biology Research Center, Zanjan Branch, Islamic Azad University, Zanjan, Iran; 3 Medical Biotechnology Research Center, School of Paramedicine, Guilan University of Medical Sciences, Rasht, Iran

**Keywords:** Divalent antibody, Flagellin, Keratitis, Mice, *Pseudomonas aeruginosa*

## Abstract

**Objective(s):**

*Pseudomonas aeruginosa* infections such as keratitis are considered among the major health problems worldwide due to the complexity of pathogenesis and antibiotic resistance crisis, thus, finding new effective approaches for prevention and treatment of the infections seem to be still vital. In this report, we aimed to investigate the therapeutic effects of topical administration of the antibodies against type a and b-flagellin (FLA and FLB) in *Pseudomonas* keratitis model of infection in mice.

**Materials and Methods:**

Scratched corneas of mice were treated with approximately 10^7^ CFUs/eye of PAK and/or PAO1 strains of *P. aeruginosa*. Specific IgG to FLA, FLB or divalent flagellin were topically applied to the infected corneas for 20 min, 24, and 36 hr post-infection. The bacterial burden and myeloperoxidase activity (as a marker for polymorphonuclears (PMNs) infiltration) were determined in the corneas. The biological activity of the anti-FLA and FLB IgG was evaluated *in vitro* by opsonophagocytosis test.

**Results:**

Compared to other treated corneas, divalent anti-flagellin IgG treatment showed a significant decrease in the bacterial CFUs and myeloperoxidase activity in the infected corneas (*P*<0.05). Results of opsonophagocytosis revealed that the specific antibodies raised against FLA and FLB had more potent opsonic killing activity on their homologous strains as compared with control group (*P*<0.05).

**Conclusion:**

It appears that in *P. aeruginosa* keratitis, topical administration of the combined antibodies likely via decreasing the bacterial load, and PMNs infiltration as well as increasing opsonophagocytosis could lead to dramatic improvement of the infected corneas.

## Introduction

Bacterial keratitis is a major cause of ocular morbidity and has become a public health concern (1). *Pseudomonas aeruginosa* (*P. aeruginosa*) keratitis is a destructive diseases of the cornea which rapidly develop. This nosocomial pathogen is a leading cause of the contact lens-related ulcerative keratitis that requires rapid diagnosis and early treatment to avoid vision loss (2). The overuse and misuse of conventional antibiotics have led to the development of multidrug-resistant (MDR) strains of *P. aeruginosa* which are difficult to overcome. Therefore, the presentation of new adjunctive prophylactic/therapeutic modalities seems to be still necessary (3).

Infection of the cornea by bacteria occurs when the epithelial barrier function becomes injured or compromised such as mild chronic damage caused by contact lens wear. In such situation, *P. aeruginosa* as an opportunistic pathogen could access to the epithelial layer and cause infection (4). Similar to other mucosal surfaces including urinary tract, airway, and intestinal, Toll-like receptor 5 (TLR5) acts as the major pathogen recognizing receptor (PRR) at the surface of the cornea (5). As the first line of defense, ocular epithelium recognizes and responds to the motile pathogen via TLR5 which activates mucosal innate defense by producing proinflammatory cytokines leading to employ polymorphonuclear leukocytes (PMNs) to the infection site. PMNs by the secretion of antimicrobial agents, directly eradicate the invading bacteria (6). The vital role of PMNs in the elimination of *P. aeruginosa* has been documented (7). TLR5-knockout mice are more susceptible to *P. aeruginosa* ocular infection (8), indicating the role of TLR5 in the forming of protective mucosal surfaces against the infection by flagellated bacteria.

Single polar flagellum-mediated motility plays an essential role in the pathogenesis of *P.* *aeruginosa *(9). This surface appendage involves in the formation of biofilm and invasion of infection. Flagellin is the major structural subunit of flagellar filament which acts as the sole ligand of TLR5. As one of the important adhesins in *P. aeruginosa*, flagellin is highly potent stimulator of host innate and acquired immunity (10). Primary attachment to the host cells is an initial and critical phase to establish an infection. Hence, passive antibody therapy against flagellin will probably reduce the bacteria attachment and eventually decline the infection (11). The flagellin (encoded by *fliC* gene) is classified into two distinct types: a-type and b-type flagellins. This classification is based on molecular weight and reactions with specific antibodies. The type a-flagellins (FLA) are a heterologous group with molecular weight of 45-52 kDa, whereas type b-flagellin (FLB) is a homologous group having a molecular weight of 53 kDa (12). Each strain only possess one type of flagellin, so that, its gene does not undergo antigenic variations. Flagellin has been utilized as a vaccine candidate against *P. aeruginosa* in different animal models: FLB protected mice in lung infection (13, 14), urinary tract infection (UTI) (15) and keratitis (16, 17). Also, immunization with FLA afforded protection in lung infections (18). Our previous work showed that active and passive immunization with FLA provided protection against *P. aeruginosa* in the burn mouse model (19). Divalent flagellin (FLA and FLB) preparation successfully confined burn wound infections (20), UTI (21) and cystic fibrosis (CF) (22). 

To our knowledge, the therapeutic effects of antibodies raised against divalent flagellin for *P. aeruginosa* keratitis has not been reported yet. In the present study, we assessed whether topical administration of antibodies to divalent flagellin (FLA and FLB) can have therapeutic efficacy in the mouse model of *P. aeruginosa* keratitis. 

## Materials and Methods

***Experimental animals***

The Female inbred BALB/c mice (weight, 20-24 g; age, 6-8 weeks) were obtained from the Pasteur Institute (Karaj, Iran). Animals were maintained in clean polypropylene cages with free access to their standard antibiotic-free diet. All animal procedures were accepted by the Institutional Animal Care and Use Committee (IACUC) of Pasteur Institute of Iran.

***Bacterial strains***

In the present research, a well-defined type b-flagellated strain of *P. aeruginosa* PAO1 (kindly provided by Dr. Abdi from Department of Microbiology, Al-Zahra University, Tehran, Iran) and a type a-flagellated strain of *P. aeruginosa* PAK (available in our laboratory) were used. The strains were controlled for purity and identified before using in this study. They were cultured in Luria Bertani (LB) broth (HiMedia, India) and maintained in 20% glycerol and kept at -20 ^°^C.

***Recombinant flagellins*** ***preparation***

As described recently, we expressed and purified the FLB as histidine-tagged proteins in a bacterial expression system (23). Briefly, for the preparation of the FLA, the *fliC* gene was obtained from *P. aeruginosa* strain HMSC05H02 (Sequence ID: KJJ22056.1). The NcoI and XhoI sites were located at the 5′ and 3′ ends of the *fliC* gene, respectively. The coding gene was designed into the expression vector pET28a to produce the recombinant pET28a/*fliC*. This recombinant vector was synthesized by Biomatik Corporation (Cambridge, Ont., Canada). The vector was transformed and expressed in *Escherchia coli* BL21 (DE3) and finally purified by Nickel-affinity chromatography. 

***Endotoxin removal***

Endotoxins or lipopolysaccharide (LPS) were eliminated from the recombinant proteins using ε-poly-L-lysine-agarose (Thermo Fisher Scientific, Inc., USA). The resin was equilibrated with Tris-HCl (10-50 mM) buffer containing 0.1-0.2 M NaCl (pH 7). The sample was applied into the resin and incubated at 4-22 °C with gentle agitation. The tube was centrifuged (500 × g for 1 min) and finally the sample was collected. To assess the presence of any remaining LPS in the samples, the Limulus amebocyte lysate assay (LAL kit, Lonza, USA) was carried out according to the manufacturer’s instructions (24).

***Murine corneal infection model***

As previously described (25), the ocular infection was initiated on injured eyes by scratch method. The PAK and PAO1 strains of *P. aeruginosa* were grown at 37 °C in Peptone Tryptic Soy Broth (PTSB, Difco Laboratories, Detroit, MI) for 18 hr and the OD_650 nm_ adjusted to inocula of about 3 × 10^7^ CFUs/eye. Female BALB/c mice anesthetized with ketamine and xylazine (Alfasan, Woerden-Holand) were placed beneath a stereoscopic microscope and then three 1-mm incisions were made on the eyes using a 26-gauge needle, and finally the bacterial strains (in a 5 μl volume) were inoculated in the injured site. The disease progression was daily monitored with a dissection microscope equipped with a digital camera.

***Preparation and administration of specific antibodies***

Two female rabbits were originally prepared from Pasture Institute (Karaj, Iran). The purified FLA and FLB were emulsified with the same volume of complete Freund’s adjuvant (Sigma, USA). The rabbits were separately immunized subcutaneously with 400 μg of the antigens at 3-week intervals. Blood samples were obtained before first immunization and 2 weeks following each immunization. The resulting sera were obtained by centrifugation (6500 × g), aliquoted (1 ml) and finally stored at -70 °C (26). The IgG rich fractions were obtained using Protein A/G agarose resin (Thermo Fisher Scientific, USA) based on the manufacturer’s protocol. The protein concentration of IgG fractions was determined by using NanoDrop spectrophotometry followed by Bradford method. Anti-flagellin IgG and non-immune rabbit serum (NRS) were stored at -20 ^°^C with final concentration of 2 mg/ml until analysis. The mice were allocated into ten different groups, each containing sixteen mice. The mice were immunized in line with the regimen described below: 

Group 1: Anti-FLA IgG (challenged with PAK)

Group 2: Anti-FLA IgG (challenged with PAO1)

Group 3: Anti-FLB IgG (challenged with PAO1)

Group 4: Anti-FLB IgG (challenged with PAK)

Group 5: Divalent IgG (challenged with PAO1 and PAK)

Group 6: NRS (control IgG)

Group 7: PBS (control group)

Group 8: Witness group (keratitis control group)

The specific IgG and control IgG fractions (NRS) were diluted in PBS to achieve their optimum concentration. The mice were anesthetized with ketamine/xylazine mixture and then specific antibodies were topically applied with 40 μg to the eyes. This was done at time 0 (approximately 20 min post-infection), 24, and 36 hr post-infection. NRS and PBS groups were challenged with PAO1 strain. Results were demonstrated as the number of cured mice in each group.

***Bacterial burden in the cornea***

To evaluate bacterial load at the infection location, the corneas (n=4/group) were taken after 24 and 72 hr after challenge with *P. aeruginosa* strains PAK and/or PAO1, and the viable bacteria were calculated using plate count technique. Individual corneas were homogenized in Tryptic soy broth including 0.5% Triton X-100, and diluted and cultured onto Cetrimide (*Pseudomonas*) agar (QUELAB, Canada) in triplicate and then incubated overnight at 37 ^°^C and bacterial colonies were enumerated (27). The results were presented as log_10_ CFU per cornea ± standard deviation (SD).

**Figure 1:**
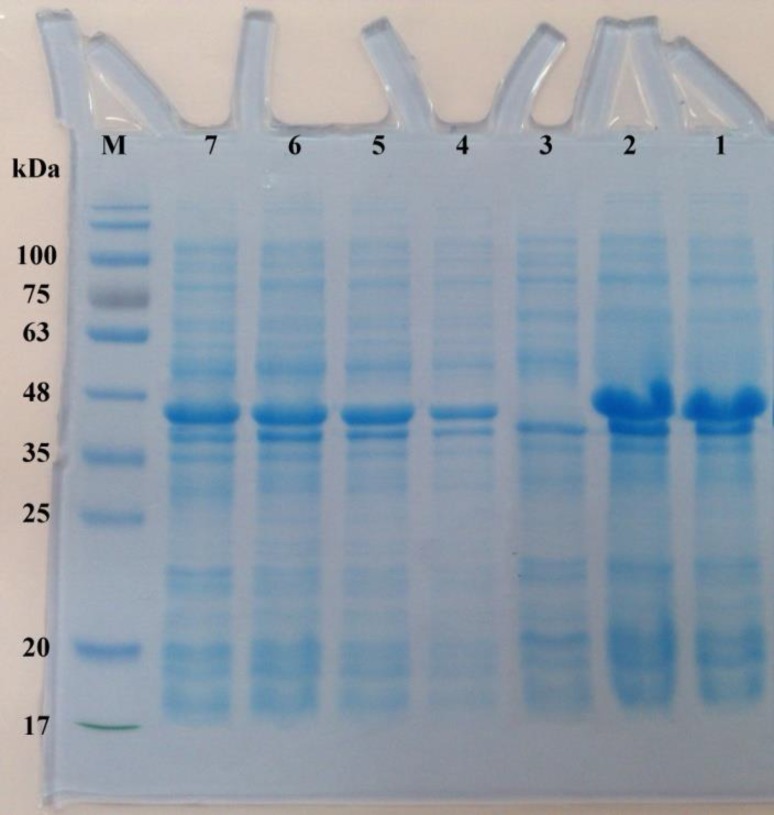
SDS-PAGE analysis of the expression of FLA protein in *Escherchia coli*. The total proteins of the *E. coli* BL21 harboring pET28a/*fliC *vector was harvested and loaded on 12% (v/v) SDS-PAGE after 4 hr induction with or without Isopropyl-β-D-thiogalactoside (IPTG). Lane M denotes marker proteins with the indicated molecular weight; (Lanes 1 and 2): purified FLA after HisTrap chelating and Ni2+-affinity chromatography (~ 45 kDa); (Lane 3): total cell lysate of non-induced bacteria; (lanes 4-7) 1-4 hr after the induction

**Figure 2:**
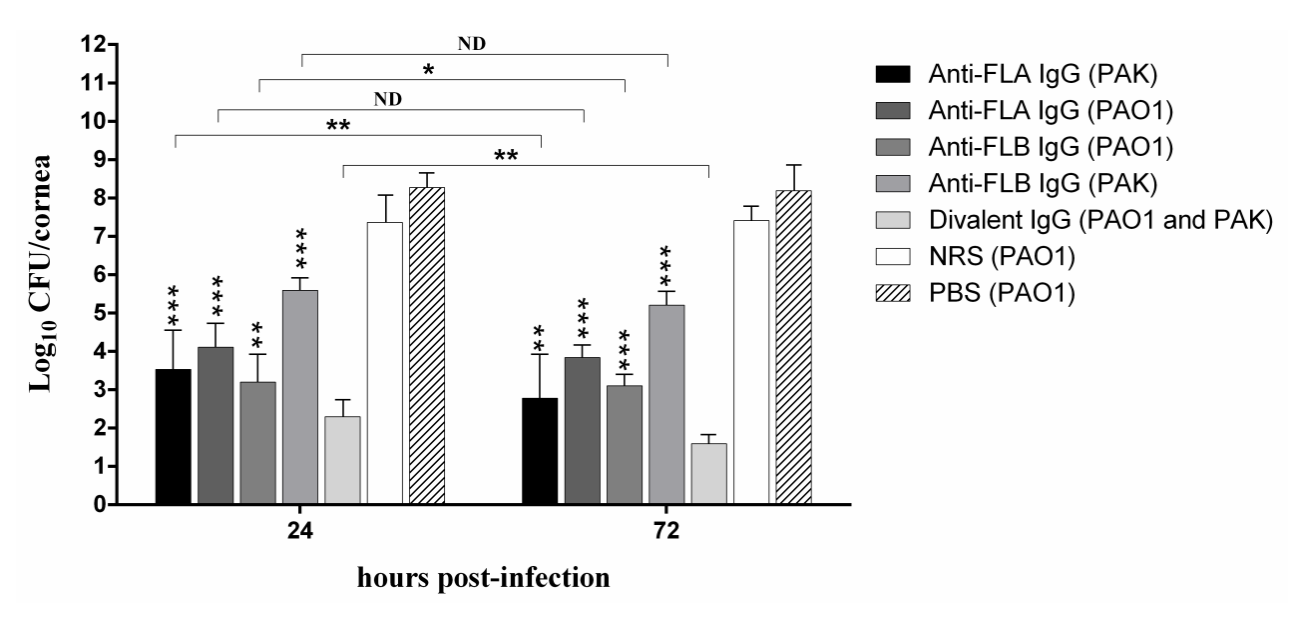
Effect of topical administration of NRS or antibodies against FLA, FLB and both on the bacterial burdens in the cornea of BALB/c mice at 24 and 72 hr post-infection. Bars represent means from duplicates of each sample, and error bars indicate the SD. Asterisk on the error bar represents significant difference between each treatment group and divalent IgG treatment group (one asterisk, *P*<0.05; two asterisks, *P*<0.01; three asterisks, *P*<0.001). ND indicates non-detectable difference

**Figure 3:**
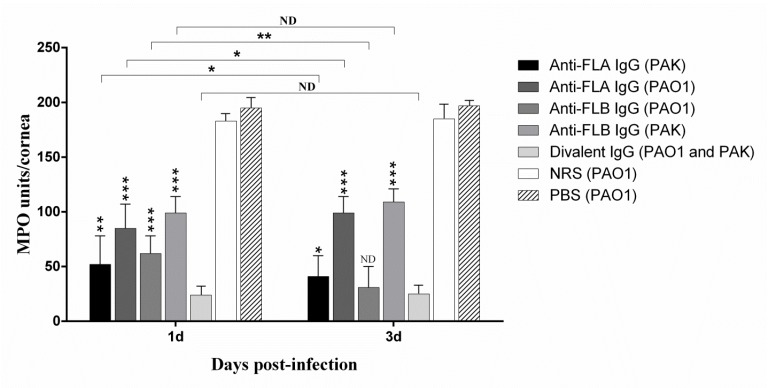
Effect of topical administration of NRS or antibodies against FLA, FLB and both on the myeloperoxidase (MPO) activity (as a marker of PMNs infiltration) in the cornea of BALB/c mice at 1 and 3 days post-infection. Asterisk on the error bar represents significant difference between each treatment group and divalent IgG treatment group (one asterisk, *P*<0.05; two asterisks, *P*<0.01; three asterisks, *P*<0.001). ND represents non-detectable difference

**Figure 4:**
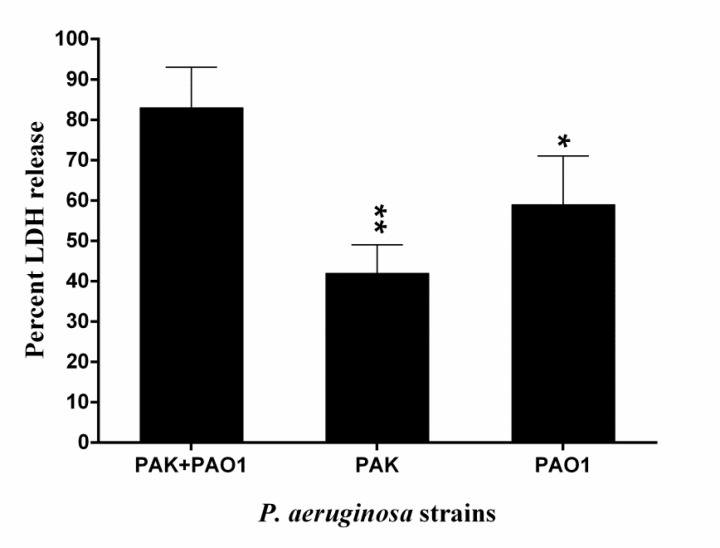
Cytotoxic activity in the infected cornea by PAK and PAO1 strains of *Pseudomonas aeruginosa*. Relative cytotoxic effect (detected by LDH release) on mouse corneal epithelium at 48 hr post-infection with PAK and PAO1 strains of *P. aeruginosa*. Bars represent means from duplicates of each sample, and error bars indicate the SD. (one asterisk, *P*<0.05; two asterisks, *P*<0.01 by comparison with “PAK+PAO1”)

**Figure 5:**
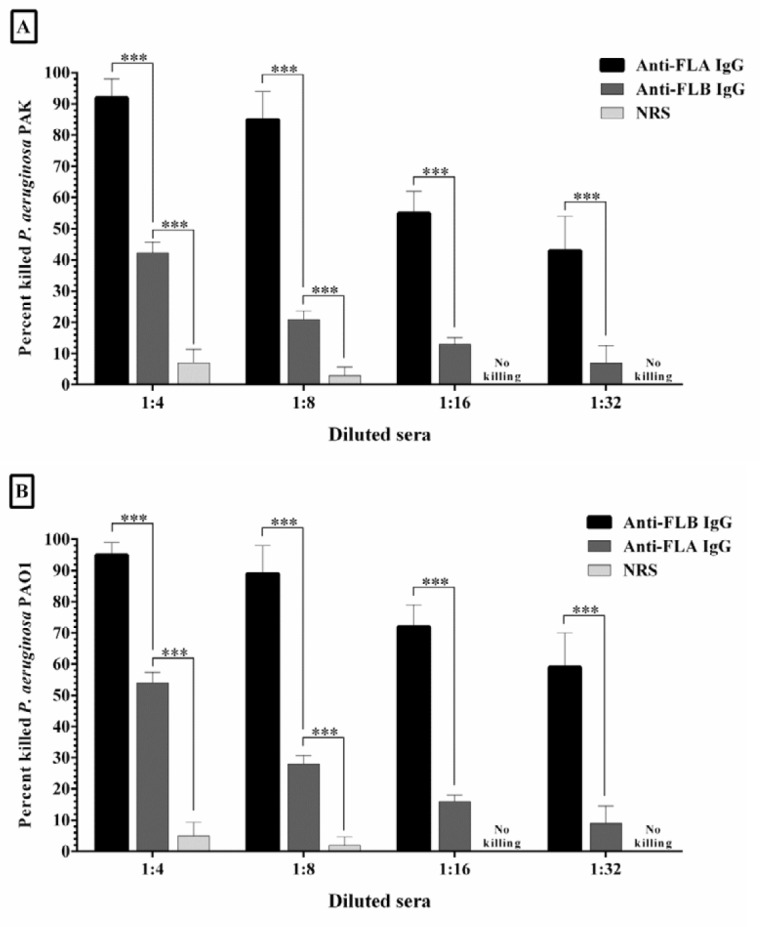
The opsonic killing activity of four two fold dilutions of specific anti-FLA and anti-FLB IgG against *Pseudomonas aeruginosa* strains PAK (A) and PAO1 (B). Each strain was separately incubated with specific antisera raised against FLA and FLB, and mouse macrophage in the presence of rabbit complement. Significant opsonophagocytosis was observed when the specific antiserum was treated with the homologous strain. It seems that the most cross-reaction was occurred when the anti-FLA IgG was treated with heterologous strain PAO1. Bars represent means of duplicate determinations, and error bars indicate SD. Three asterisks represent *P*<0.001

***PMN infiltration assay***

Myeloperoxidase (MPO) activity in the cornea as a marker of PMN infiltration was measured according to the previously described method (28). Briefly, one and three days post-infection, the corneas (n=4/group) were homogenized in hexadecyltrimethylammonium bromide (HTAB) buffer and then subjected to three freeze-thaw cycles, followed by centrifugation. Each supernatant was combined with phosphate buffer containing O,O-dianisidine hydrochloride and hydrogen peroxide to obtain a total volume of 3 ml. The absorbance changes at 460 nm were continuously monitored for 5 min. The results were presented as units of MPO activity/cornea. One unit of MPO activity was corresponded to about 2 × 10^5^ PMNs (29).

***Corneal cell cytotoxicity***

48 hr post-infection, extracellular lactate dehydrogenase (LDH) was determined as a marker of *P. aeruginosa*-induced cell damage according to the described earlier (30). Briefly, the corneas (n=4/group) were scratched and infected with approximately 10^7^ CFUs of *P. aeruginosa* strains PAK and/or PAO1 for 48 hr. To measure the extracellular LDH, the infected corneas were collected and washed with PBS including PMSF (as protease inhibitor). By using a cytotoxicity detection kit plus (Roche Diagnostics, Mannheim, Germany), the extracellular LDH release was detected.

***Opsonophagocytic killing assay***

The *in vitro* opsonophagocytosis test was done according to the protocol of Faezi *et al* (23). In brief, 100 µl of bacterial inoculum [~ 2 × 10^9^ CFUs/ml of *P. aeruginosa* PAK or PAO1 in 1% bovine serum albumin (BSA)] was exposed to the same volume of heat-inactivated four two fold dilutions (1:4 to 1:64) of specific anti-FLA and anti-FLB IgG at 22 ^°^C for 60 min. Then, it was washed two times with BSA [1% (w/v)] to remove excessive antibodies. One hundred µl of mouse macrophages [~ 2 × 10^7^ CFUs/ml in RPMI-1640 supplemented with 10% heat-inactivated fetal bovine serum (FBS)] and 100 µl of 10% infant rabbit serum (complement source) were added to the mixture and incubated for 90 min at 37 ^°^C. The control samples were run with each assay by excluding antibody, complement or macrophages. Eventually, a 100 µl of aliquot was collected, diluted in PBS, and plated for bacterial count. The opsonophagocytosis activity of the sera was determined by following formula: 

[1 - (CFU immune serum at 90 min/CFU of pre-immune serum at 90 min)] × 100

***Statistical analysis***

Results are presented as mean±SD. Comparisons between groups were done by using the one-way analysis of variance (ANOVA) followed by *post hoc* Tukey’s test. In each group, Paired t-test was used to compare between 24 and 72 hr. For all tests, *P*<0.05 was considered as statistically significant. Results were analyzed using Graph Pad Prism v6.01 software (Graph Pad Software, San Diego, CA, USA).

## Results

***Overexpression and purification of the FLA***

The coding sequence of FLA (*fliC* gene) was constructed into the pET28a to express a C-terminal His-tagged protein. The recombinant vector pET28a/*fliC* was transformed into *E. coli* BL21 (DE3). The SDS-PAGE analysis revealed that the highest amount of FLA was produced by induction with 1 mM Isopropyl-β-D-thiogalactoside (IPTG; as inducer) at 37 ^°^C for 4 hr. The expression product of the FLA protein was at a molecular size of approximately 45 kDa (Figure 1). After stepwise dialyses, 3.89 mg renatured FLA was obtained from 1 l of the culture. By using quantitative LAL kit, the LPS quantity in the FLA preparation was 0.0072 endotoxin unit/μg protein. The concentration of anti-FLA and anti-FLB IgG fractions was monitored using Bradford and NanoDrop spectrophotometer, which revealed the approximate values 13.7 and 15.1 mg/ml, respectively. 

***Bacterial burden in the cornea of different experimental groups***

We evaluated whether antibody to divalent flagellin has therapeutic efficacy on PAK and PAO1 strains of *P. aeruginosa* in the ocular infection. We applied the antibody topically to the surface of the infected corneas and viable bacteria extracted from the corneas were determined at 24 and 72 hr post-infection (see Figure 2). The polyclonal rabbit IgG to divalent flagellin yielded a significant reduction in the bacterial load of the cornea 24 hr after infection with PAK and PAO1 strains compared to the other experimental groups (*P* <0.05). The number of viable bacteria recovered from the divalent anti-flagellin IgG group was at least 1 log less than other groups. Also, at 3 days post-infection, the divalent IgG group exhibited fewer bacteria in the cornea than other treatment and control groups (*P*<0.05) and even than the cornea 1 day post-infection (*P*=0.0066). We have also demonstrated that topical application of anti-FLA IgG significantly reduced the bacterial load in the cornea 72 hr after infection with the homologous PAK strain in comparison with the bacterial load 24 hr post-infection (*P*=0.0092). However, the anti-FLA IgG showed some cross-reactivity with PAO1 strain, so that diminished the number of the heterologous strain from the cornea (*P*=0.069). At 72 hr post-infection with strain PAO1, antibody against FLB led to significant decrease in the bacterial CFUs compared to that of 24 hr after infection (*P*=0.042); however, a slight therapeutic effect was observed on the heterologous strain PAK (*P*=0.075). No significant difference in bacterial burden was determined between the NRS and PBS groups even after 72 hr post-infection (*P*=0.094) (Figure 2).

***PMNs infiltration in the cornea of different experimental groups***

We evaluated if PMNs were participated in enhanced bacterial clearance in the immunized cornea. So, the bioactivity of MPO in the cornea of ​​different experimental groups was measured as a marker of PMNs infiltration. Compared to the other treated corneas, divalent anti-flagellin IgG treatment group exhibited significantly lower MPO activity in the infected corneas (*P*<0.05) (Figure 3). Little more leukocytes MPO activity was observed in the two groups immunized and infected with the homologous strain as compared with divalent IgG treated group. There was high detectable MPO activity in the PBS and NRS groups (Figure 3), this finding is in line with the differences in bacterial load of the infected mouse eyes (Figure 2) wherein the lowest bacterial load was observed in the mice immunized with divalent IgG. No statistically significant difference was observed between one day and 3 days post-infection in the divalent IgG treated group (*P*=0.072). 

***P. aeruginosa-induced*** ***cytotoxicity in the*** ***infected cornea ***

We assessed the cytotoxic effects of two strains of *P. aeruginosa* on the mouse corneas. The corneas co-infected by PAK and PAO1 strains showed a higher level of extracellular LDH in comparison with those infected with PAK or PAO1 strain separately (*P*=0.0047 and *P*=0.028, respectively) (Figure 4). 

***Opsonophagocytic killing activity***

Antibody-mediated phagocytosis means the engulfment of the opsonized bacteria with phagocytes which is correlated with the clearance of infection. In this test, specific rabbit IgG raised against FLA and FLB separately incubated with PAK and PAO1 strains of *P. aeruginosa*. A significant killing was observed when the anti-FLA IgG and anti-FLB IgG incubated with their homologous strain in a 1:4 dilution (92.1% and 95.3% in comparison with control group after 90 min, respectively). Less than 6.7% opsonic activity was detected by NRS, which is most likely indicating the non-opsonic phagocytosis. As expected, there was a decrease in the opsonophagocytosis activity with the increased dilution of the antisera, although this slope was steeper when anti-FLA IgG was treated with homologous PAK strain. We demonstrated that the antibodies raised against purified FLA had medium opsonic activity (54%) on the heterologous PAO1 strain. Also, some cross-reaction (42.3%) was observed between anti-FLB IgG and heterologous PAK strain (Figure 5). 

## Discussion

Flagellin is one of the major pathogen-associated molecular pattern (PAMP) in Gram-negative bacteria, and participates in the initial attachment of *P. aeruginosa* to the host epithelial cells. It appears that the specific anti-flagellin antibodies by decreasing the attachment and motility of the bacteria could control the infection. In this report**,** we revealed a new therapeutic role of antibodies raised against divalent flagellin (FLA and FLB) in the mouse model of *P. aeruginosa *keratitis. In the present study, we utilized a well-known and accepted mouse model of *P. aeruginosa *keratitis (25). We showed that the mouse corneal tissue infected with PAK and PAO1 strains of *P. aeruginosa*, released higher levels of LDH compared to that of infected with PAK or PAO1 alone. This result indicates that co-infection with these two strains is more cytotoxic than infection by each alone. 

We also found that following passive immunization of the infected mice with divalent IgG, a vast elimination of the bacteria at an early stage of the infection occurred, which might be due to the presence of strong innate immune defenses in the cornea. We demonstrated that the treatment of infected mice with divalent IgG resulted in a four fold reduction in the viable bacteria in the cornea at 1 day post-infection and six fold at 3 days post-infection compared to other experimental groups. The topical administration of anti-FLA or anti-FLB IgG considerably reduced the bacterial burden in the cornea after infection with their homologous strain (PAK and PAO1, respectively) and had medium cross-reactivity with their heterologous strain (PAO1 and PAK, respectively). The specific IgGs to both types of the flagellins could lead to a significant elimination of *P. aeruginosa *in the infected corneas after infection. In the study of Kumar *et al*. (28) topical administration of FLB through subconjunctival way (100 ng/eye) 24 hr before the *Pseudomonas* infection remarkably decreased the bacterial burden in the cornea of the B6 mice. So, the protective role of the flagellin administration as TLR5 inducer and anti-inflammatory approach was proposed (28, 31). Irrespective of the differences in the study design, it appears that divalent flagellin IgG may be more effective therapeutic approach to control inflammation and corneal injury caused by the opportunistic pathogen. 

We also showed the reduction in the MPO activity in divalent IgG-treated corneas at the late stage of infection, suggesting that this treatment approach induced low resolution of the inflammation. It seems that remarkably reduction in inflammation and tissue damage of divalent IgG-treated BALB/c mice corneas is associated with the enhanced bacterial clearance. It has been shown that *P. aeruginosa *infection evokes a great increase in antimicrobial peptides (AMPs) expression such as cathelin-related antimicrobial peptide (CRAMP), which act as a potential innate defense mechanism in the cornea (32, 33). The increased bacterial clearance induced by divalent IgG is partly related to the induced AMPs gene expression by unaffected lower PMN infiltration in the corneas by the bacteria. It appears that the low levels of infiltrated PMNs in anti-FLA IgG (PAK challenged), anti-FLB IgG (PAO1 challenged) and divalent IgG treated corneas at 3 day after infection might be resulting from reduction in bacterial load and *P. aeruginosa*-induced high expression of AMPs like CRAMP. Some reports have also demonstrated that bacterial flagellin could cause dendritic cells (DCs) activation (one of the major member of innate immune defense) (34, 35) as well inducible nitric oxide synthase (17, 36). In our case, the presence of the flagellated strains of *P. aeruginosa* on injured corneas may also induce the activation of DCs. These findings were to somewhat in line with the previous studies in which the administration of flagellin (225 ng per mouse) through subconjunctival and intraperitoneal injection 24 hr before bacterial infection led to preserved structural integrity of the cornea and a significant decrease in PMNs infiltration within the cornea, consequently resulted in effective prevention of *P. aeruginosa* keratitis (28, 31). 

Antibody-mediated opsonophagocytosis is an innate immune defense which involves antibody binding to the bacterial antigen. The neutralized bacteria are engulfed by macrophages and DCs, eventually leading to the clearance of the infection. Following the investigation of opsonophagocytic activity of anti-FLA and anti-FLB IgG on *P. aeruginosa*, we concluded that the specific antibodies had significantly high opsonic killing activity on their homologous strains, indicating the type-specific action of the antisera. These antisera also induced phagocytosis of their heterologous strains to some extent, this indicates moderately similarity of the amino acid composition between FLA and FLB (13). These results were in contrast to Campodonico *et al. *study where anti-FLA antibody had low opsonophagocytic activity against homologous PAK strain and no killing activity on the PAO1 strain, and anti-FLB had no opsonophagocytic activity against either homologous or heterologous strains (13). These dissimilarities might be partially attributed to the different dose of the used flagellin for rabbit immunization or heterogeneity of the flagellin (13, 24, 37). Altogether, the combined effects of antibody-mediated opsonophagocytosis partly attributed to the significant enhance in bacterial clearance at the early stage of the infection, resulting in improvement in the consequence of infection in the injured cornea. 

## Conclusion

The presence of specific IgGs against both types of flagellin could enhance the therapeutic efficacy against possible presence of both type a and b-flagellated strains in the cornea. It seems that topical application of IgG to divalent flagellin in the infected cornea could significantly reduce the bacterial burden and most strikingly, increase antibody-mediated opsonophagocytosis, as well as decrease PMNs infiltration post-infection, leading to the preservation of corneal function in the BALB/c mice. Considering the fact that most of the bacterial infections in the cornea take place only when the epithelial barrier is injured (e.g., especially in individuals wearing contact lenses), the addition of divalent IgG at the ocular surface through contact lens solutions or eye drops may have prophylactic effects for *in situ* prevention of contact lens-related corneal infection. Further studies to evaluate the efficacy and safety of the topical treatment are necessary. Ongoing studies by our teamwork are addressing the specific role of the active immunization with divalent flagellin to prevent both flagellin-expressing strains of *P. aeruginosa* (dual targeting) in the keratitis model of infection in mice.
